# *Notes from the Field*: Invasive Group G β-Hemolytic *Streptococcus* Outbreak at a Long-Term Care Facility — Pennsylvania, 2024

**DOI:** 10.15585/mmwr.mm7434a2

**Published:** 2025-09-11

**Authors:** Monica Giacomucci, Laxmi Modali, Cara Bicking Kinsey, Kim Warren, Sopio Chochua, Christopher J. Gregory, Allison Longenberger

**Affiliations:** ^1^Pennsylvania Department of Health, Bureau of Epidemiology; ^2^Division of Bacterial Diseases, National Center for Immunization and Respiratory Diseases, CDC.

**Summary**

**What is already known about this topic?**

Group G β-hemolytic *Streptococcus* (GGS) is increasingly recognized as a cause of invasive disease but is not reportable in Pennsylvania. GGS outbreaks in long-term care facilities (LTCFs) have not been reported.

**What is added by this report?**

Two patients aged >85 years who were residents of an LTCF developed GGS bacteremia; one died. Two other residents had positive wound cultures and were treated with antibiotics. Genomic analysis suggested isolates were highly related. Multiple infection control breaches were identified.

**What are the implications for public health practice?**

Public health response tools developed for outbreaks of group A *Streptococcus* in LTCFs, including infection control assessment and colonization screening, were successfully applied to control this outbreak of GGS. Public health monitoring for GGS might help detect similar clusters in LTCFs.

In November 2024, the Pennsylvania Department of Health (PADOH) was notified of two β-hemolytic group G *Streptococcus* (GGS)–positive blood culture results from residents of the same long-term care facility (LTCF) (a skilled nursing facility) who were receiving wound care. Both patients were hospitalized for sepsis and cellulitis; one patient died. Clinical presentation and GGS-positive blood cultures without other pathogens detected supported a diagnosis of invasive GGS. GGS, a normal commensal organism, is increasingly recognized as a cause of invasive disease secondary to soft tissue infections, including cellulitis (*1*,*2*). Group A *Streptococcus* (GAS) is known to cause invasive disease in patients with soft tissue infections; LTCF GAS outbreaks are well documented, and response tools are available (*3*). However, whereas invasive group A *Streptococcus* infections are monitored through ongoing surveillance in many parts of the country and are reportable in Pennsylvania, invasive GGS infections are not (*1*,*2*). Although GGS transmission modes and clinical presentation are thought to be comparable to those of GAS (*1**,**2*,*4*), the epidemiology and clinical characteristics of GGS infections in LTCF are not well described. During December 2024, PADOH conducted a facility site visit. It used the standard GAS protocol to describe patient characteristics; observe infection prevention and control (IPC) practices; conduct colonization screening; and provide prevention recommendations. The PADOH Institutional Review Board determined that the activity met the criteria for public health surveillance and therefore did not constitute research. This activity was reviewed by CDC, deemed not research, and was conducted consistent with applicable federal law and CDC policy.*

## Investigation and Outcomes

### Patient Characteristics

Both patients, women aged >85 years, had underlying medical conditions. Patient A, a resident at the facility since 2021, had long-term bladder catheter use and cellulitis; after her hospitalization, she returned to the facility. Patient B, living at the facility since October 2024, had heart failure, multiple myeloma, chronic deep vein thrombosis, and cellulitis; patient B died 1 day after her acute hospital admission.

### Assessment of IPC Practices

Review of facility IPC policies and practices and observation of wound care and hand hygiene identified numerous protocol breaches. The facility’s hand hygiene policy did not specify a preference for use of alcohol-based hand sanitizer in a majority of clinical situations, which differs from CDC guidance. Successful hand hygiene^†^ was observed during 22 (50%) of 44 instances during which hand hygiene was indicated. Observation of wound care among 13 residents identified infection control breaches^§^ during all 13 occurrences, including not preparing a clean field before a procedure, improper handling^¶^ of multidose topical medications, and moving from dirty to clean tasks without performing hand hygiene. PADOH provided written IPC and surveillance recommendations, based on experience with and guidelines for investigating and controlling GAS infections in LTCFs (*3*).

### Identification of Colonization

In January 2025, using GAS guidelines (*3*), all residents receiving wound care (12 [17%] of 70, excluding patient A, who had returned to the facility) were screened for GGS colonization; throat swabs were collected from all 12 residents, and 15 wound swabs were collected from nine residents.** Throat swabs were also collected from the two staff members who provided wound care. Culture-based testing by the state public health laboratory identified two colonized patients through positive wound swab culture results; all throat swab test results were negative. In response to CDC and PADOH recommendations for GAS decolonization (*3*), a 10-day course of oral cephalexin was provided to the colonized residents. Repeat testing 30 days after starting antibiotics remained positive for both residents; one resident received a 10-day course of oral ampicillin, the second received a 10-day course of oral ciprofloxacin. Wound cultures were negative for both on subsequent testing.

### Whole Genome Sequencing of Isolates

Four isolates (two blood culture isolates from patients A and B and two obtained from resident wound colonization screening) were sent to CDC for *emm* typing^††^ and whole genome sequencing. All were *emm* type 2574.3 and previously uncharacterized multilocus sequence type 525; the high relatedness of the strains (1–2 single nucleotide polymorphism differences) suggests a common source.

## Preliminary Conclusion and Actions

In an English-language literature search of PubMed using keywords “group G *Streptococcus,” *“*Streptococcus dysgalactiae *subsp.* equisimilis,”* and “long-term care facility,” no previous reports of an outbreak of invasive GGS at a LTCF in the United States were identified. High genomic relatedness among clinical and colonization isolates suggests intrafacility transmission, likely resulting from suboptimal IPC practices. The epidemiologic characteristics, outcomes, and patient risk factors identified in this investigation were similar to those observed in GAS outbreaks, including advanced patient age, chronic comorbidity, the presence of wounds, colonization of residents who share health care staff members, and severe outcomes among infected patients (*3*,*5*). In the absence of established guidance for GGS outbreak response in LTCFs, PADOH followed GAS guidance (*3*). No additional GGS infections have been reported by the facility. Jurisdictions might consider including GGS clusters in routine surveillance protocols. The public health response tools for GAS can likely be applied to outbreaks involving other groups of β-hemolytic *Streptococcus* in LTCFs.

## Footnotes

* 45 C.F.R. part 46.102(l)(2), 21 C.F.R. part 56; 42 U.S.C. Sect. 241(d); 5 U.S.C. Sect. 552a; 44 U.S.C. Sect. 3501 et seq.

† A successful hand hygiene moment is defined as performance of hand hygiene using correct technique (either by washing the hands with soap and water or by using alcohol-based hand rub) before, during, or after a patient interaction in which hand hygiene is indicated by CDC.

§ A breach in wound care is defined as an occurrence during a wound care procedure wherein the health care provider does not follow infection control best practices. Council for Outbreak Response: Healthcare-Associated Infections and Antimicrobial-Resistant pathogens | Framework for healthcare-associated infection outbreak notification | 2022

¶ Whenever possible, multidose medication containers (e.g., creams, sprays, or ointments) should be dedicated to a single resident; if not possible, then a small amount should be allocated for each resident. Medication containers should not be taken into resident rooms.

** Three residents from whom throat swabs were collected refused collection of a wound swab.

†† Sequence analysis of part of the M protein gene, which encodes the cell surface M virulence protein.
